# Selection, diversity and evolutionary patterns of the MHC class II DAB in free-ranging Neotropical marsupials

**DOI:** 10.1186/1471-2156-9-39

**Published:** 2008-06-05

**Authors:** Yvonne Meyer-Lucht, Celine Otten, Thomas Püttker, Simone Sommer

**Affiliations:** 1 Evolutionary Genetics, Leibniz Institute for Zoo and Wildlife Research, Alfred-Kowalke-Str. 17, D-10315 Berlin, Germany; 2 Animal Ecology and Conservation, Biozentrum Grindel, Department of Biology, University of Hamburg, Martin-Luther-King-Platz 3, D-20146 Hamburg, Germany

## Abstract

**Background:**

Research on the genetic architecture and diversity of the MHC has focused mainly on eutherian mammals, birds and fish. So far, studies on model marsupials used in laboratory investigations indicated very little or even no variation in MHC class II genes. However, natural levels of diversity and selection are unknown in marsupials as studies on wild populations are virtually absent. We used two endemic South American mouse opossums, *Gracilinanus microtarsus *and *Marmosops incanus*, to investigate characteristic features of MHC selection. This study is the first investigation of MHC selection in free-ranging Neotropical marsupials. In addition, the evolutionary history of MHC lineages within the group of marsupials was examined.

**Results:**

*G. microtarsus *showed extensive levels of MHC diversity within and among individuals as 47 MHC-DAB alleles and high levels of sequence divergence were detected at a minimum of four loci. Positively selected codon sites were identified, of which most were congruent with human antigen binding sites. The diversity in *M. incanus *was rather low with only eight observed alleles at presumably two loci. However, these alleles also revealed high sequence divergence. Again, positive selection was identified on specific codon sites, all congruent with human ABS and with positively selected sites observed in *G. microtarsus*. In a phylogenetic comparison alleles of *M. incanus *interspersed widely within alleles of *G. microtarsus *with four alleles being present in both species.

**Conclusion:**

Our investigations revealed extensive MHC class II polymorphism in a natural marsupial population, contrary to previous assumptions. Furthermore, our study confirms for the first time in marsupials the presence of three characteristic features common at MHC loci of eutherian mammals, birds and fish: large allelic sequence divergence, positive selection on specific sites and trans-specific polymorphism.

## Background

The vertebrate immune system possesses two very efficient tools to ward off constantly evolving pathogens, the innate and the adaptive immune system. As a part of the adaptive immune system the molecules of the major histocompatibility complex (MHC) recognize antigens, present them to T-lymphocytes and thereby initiate an immune response [1]. The need to recognize a wide range of pathogens drives an adaptive polymorphism in the MHC, which indeed contains the most variable functional genes in vertebrates [2,3]. Therefore, the MHC constitutes a powerful model to study processes, causes and consequences of selection on a molecular level [3-5].

The MHC is a multigene family that codes for cell-surface glycoproteins. These molecules are key receptors for the presentation of peptide fragments deriving from pathogens. MHC class I molecules mainly correspond to intracellular pathogens and are expressed on the surface of all nucleated somatic cells. MHC class II genes are predominantly involved in the defence against extracellular pathogens and are expressed only on specialized antigen-presenting cells, such as B cells and macrophages [6]. The polymorphism at the MHC class I and II genes is especially pronounced in the codons that are directly involved in antigen binding, the so-called antigen binding sites (ABS) [7,8].

Research on the genetic architecture and diversity of the MHC has focused mainly on eutherian mammals, birds and fish. From this broad range of studies it has become apparent that the extreme allelic diversity found at class I and II loci is characteristic for the MHC [4]. This unusually high level of polymorphism found at the MHC of most vertebrate species is assumed to be maintained by means of balancing selection. It is supposedly driven by pathogens through heterozygote advantage and/or frequency-dependent selection [3,5,9-11] as well as mechanisms linked to reproduction, such as disassortative mating or pre-natal selection [12-14]. Balancing selection is reflected in an increased rate of non-synonymous over synonymous substitutions and an elevated rate of recombination events relative to neutral expectations as well as in retaining certain alleles longer than expected under a neutral model [7,8,15,16].

The latter phenomenon has been described as trans-species polymorphism [17], where certain MHC alleles or allelic lineages are found in related species indicating that they are older than the speciation event and passed on from the ancestral to the descendant species. Long-lasting trans-species polymorphism occurs only in genetic systems under balancing selection and is a typical mode of evolution in the MHC [18].

In contrast to the great attention being paid to the MHC of eutherian mammals, birds and fish, the diverse group of marsupials has been poorly investigated so far. Earlier studies on marsupial cellular immunology reported dramatic differences in the immune response between marsupials and eutherians and predicted little or no polymorphism at the MHC class II genes [19-21]. Only recently has the complete MHC region been mapped for the first marsupial (*Monodelphis domestica*) [22,23]. Belov et al. [24] presented in a phylogenetic review that the marsupial MHC class II β genes cluster in two groups clearly separated from the eutherian β gene families, and therefore constitute non-orthologous loci. Recently, a third lineage has been reported [22]. The marsupial and eutherian lineages appear to have maintained different MHC class II β gene clusters after duplication events early in the mammalian evolution. The authors recommended using the nomenclature DAB, DBB and DCB for the marsupial β gene families [22,24].

At present, molecular research on the marsupial MHC class II is restricted mainly to captive or laboratory bred individuals. So far five marsupial species have had their MHC class II β genes examined. Regarding the Australian marsupials, Schneider and co-workers [25] investigated one red-necked wallaby (*Macropus rufogriseus*) from a zoo and detected two DAB alleles, one of them a transcribed pseudogene, and one DBB allele. A study on the MHC of captive bred tammar wallabies (*Macropus eugenii*) using RFLP suggested at least 12 MHC class II β loci in tammars, but variation between individuals appeared to be significantly reduced compared with most eutherians [26]. In *Trichosurus vulpecula*, the brushtail possum, five DAB alleles were discovered, again from a single individual [27]. Siddle et al. [28] isolated six MHC II DAB alleles from one Tasmanian devil (*Sarcophilus harrisii*), and very recently a study on wild 26 individual *S. harrisii *revealed no additional alleles [29]. In the sole South American marsupial to have its MHC studied, *M. domestica *(gray short-tailed opossum), only a single DAB sequence was identified from five individuals [30], and two DBB and one DCB sequences in a genome sequencing project [22].

Levels of natural diversity and processes of selection on the marsupial MHC are largely unknown, as studies on wildlife population of marsupials virtually do not exist [but see [29]]. Yet, marsupials represent an important milestone in the mammalian evolution. Based on genetic data the separation of marsupials from eutherians took place some 120 to 100 million years ago [31]. Tracing back the evolution of the adaptive immune system in mammals may allow fundamental insights to its origin and function.

The marsupials of South and Central America are a widespread group comprising 76 recent species [31]. There are species adapted for dry habitats or cool regions at high altitudes of the Andes, but the majority of American marsupials are rainforest dwellers [31]. As representatives of free-ranging marsupials under natural condition, we chose two species of mouse opossums that belong to the largest family of American marsupials, the Didelphidae: *Gracilinanus microtarsus *(Brazilian gracile mouse opossum, Wagner 1842) and *Marmosops incanus *(Gray slender mouse opossum, Lund 1840). These two species are endemic to the highly threatened coastal Atlantic forest of South America [32,33] and differ in their sensitivity to habitat fragmentation [34,35], one of the major threats to the coastal Atlantic forest. By studying these two species we aimed to (1) investigate MHC selection processes for the first time in free-ranging populations of marsupials, and (2) test whether the predicted low levels of polymorphism at MHC class II in marsupials are to be confirmed under natural conditions. Moreover, in a phylogenetic comparison we explored (3) the evolutionary history (trans-species evolution) of MHC lineages within marsupials comprising *G. microtarsus*, *M. incanus *and additional marsupial taxa.

To the best of our knowledge this is the first study on natural levels of MHC diversity in non-model, free-ranging Neotropical marsupials.

## Results

### MHC-DAB diversity in *G. microtarsus *and *M. incanus*

The MHC-DAB genes of 54 Brazilian gracile mouse opossums (*G. microtarsus*) and 56 Gray slender mouse opossums (*M. incanus*) were genotyped. Following the suggestion of Klein et al. [36], the DAB alleles of *G. microtarsus *and *M. incanus *were denominated as *Grmi*-DAB and *Main*-DAB, respectively. A BLAST search revealed >80% identity of all identified *Grmi*-DAB and *Main*-DAB alleles to MHC II β genes of the gray short-tailed opossum (*M*. *domestica*), the tammar wallaby (*M*. *eugenii*), the brushtail possum (*T*. *vulpecula*) or eutherian DRB sequences. The alleles discovered in this study were published in GenBank under accession No's EU350142 – EU350149 and EU350150 – EU350196.

**Figure 1:**
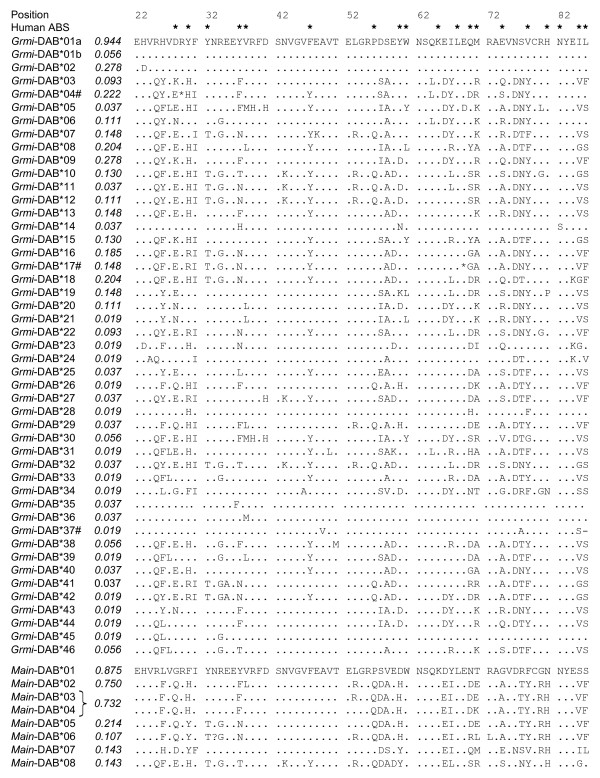
**Alignment of amino acid sequences of *Gracilinanus microtarsus *MHC-DAB alleles (*Grmi*-DAB) and *Marmosops incanus *MHC-DAB alleles (*Main*-DAB)**. Allele frequencies are given in italic, dots represent sequence identity with allele *Grmi*-DAB*01a and # indicates a pseudogene. The bracket combining *Main*-DAB*03 and *Main*-DAB*04 signifies that these two alleles revealed identical SSCP patterns and the frequency value is therefore composed from both alleles. Numeration is according to the human DR1 molecule and asterisks indicate the human antigen binding sites (ABS) defined by [37].

*G. microtarsus *displayed a high degree of MHC-DAB diversity, both within and among individuals (Table 1, Fig. 1). A total of 47 distinct *Grmi*-DAB alleles were discovered through cloning and sequencing of fifteen recombinant clones per individual (Fig. 1). The average number of alleles per individual was 4.6 ± 1.7, and ranged from one to seven; thus we report a minimum of four DAB loci in *G*. *microtarsus*. The alleles *Grmi*-DAB*04 and *Grmi*-DAB*17 contained stop codons, and one allele (*Grmi*-DAB*37) carried a deletion of two nucleotides that lead to a frameshift. These three alleles were classified as pseudogenes and excluded from further analyses. In the remaining 44 nucleotide sequences, 93 out of 195 positions were variable (Table 1). The alleles differed in one to 46 nucleotide positions, with an average of 28.2 ± 2.6. On the amino acid level 44 out of 65 positions were polymorphic. Two nucleotide sequences translated to the same amino acid sequence (*Grmi*-DAB*01a and *Grmi*-DAB*01b). Except for these two, alleles differed in one to 26 positions, with an average of 15.9 ± 2.1. The most common allele *Grmi*-DAB*01a was detected in all but three individuals (frequency= 0.944), followed by *Grmi*-DAB*02 and *Grmi*-DAB*09 (both 0.278). The remaining *Grmi*-DAB alleles occurred in one to twelve individuals (Fig. 1).

**Table 1:** MHC-DAB diversity in *G. microtarsus *and *M. incanus*

Species	N	DAB alleles	DAB loci	Nucleotide sequence		Amino acid sequence	
		
				Variable positions	Ø differences	Variable positions	Ø differences
*G. microtarsus*	54	44 (+3)	≥ 4	93/195 (47.7%)	28.2 ± 2.6 (14.5%)	44/65 (67.7%)	15.9 ± 2.1 (24.5%)
*M. incanus*	56	8	≥ 2	66/195 (33.8%)	25.6 ± 2.7 (13.1%)	30/65 (46.2%)	14.1 ± 2.2 (21.7%)

The number of DAB alleles (+ sequences including a stop codon or a frameshift) in the samples (N) and the inferred minimum number of DAB loci are displayed for both species, as well as variable positions and the mean number of differences between alleles (Ø differences).

Unlike *G. microtarsus*, the number of different DAB alleles found in *M. incanus *was low (Table 1). A total of eight *Main*-DAB alleles were assigned via SSCP and cloning from 56 individuals (Fig. 1). The number of alleles within an individual was on average 3.0 ± 0.6, with a range from two to four alleles. Hence in *M. incanus *the DAB locus is at least duplicated. Nucleotide divergence between the *Main*-DAB alleles was high. In eight *Main*-DAB alleles, 66 from 195 nucleotide positions were variable and no indels were detected. Alleles differed amongst themselves by two to 45 nucleotide positions and in 25.6 ± 2.7 on average. Each nucleotide sequence translated to a unique amino acid sequence. 30 out of 65 amino acid positions were polymorphic. Alleles varied in one to 24 positions, averaging in 14.1 ± 2.2 substituted amino acids. The most common allele *Main*-DAB*01 was present in a frequency of 0.875, followed by *Main*-DAB*02 (0.750) and *Main*-DAB*03/4 (0.732). The remaining alleles occurred in ≤ 12 individuals (Fig. 1).

### Test for positive selection

The marsupial MHC-DAB was suggested to be not orthologous to the eutherian DRB [24], therefore we did not assume an *a priori *concordance with the antigen binding sites from the human HLA class II molecule DR1 [37,38]. Accordingly, the *d*_*N*_/*d*_*S *_ratio, which is commonly applied to test for balancing selection [7,8], was calculated for the whole sequence and not separately for the ABS and non-ABS. There was no excess of non-synonymous over synonymous substitutions for the whole sequence in both species (Table 2). However, in alignment with the human DR1 molecule, 17 (89%) positions in *G. microtarsus *and 15 (79%) positions in *M. incanus *out of the 19 predicted ABS revealed variation (Fig. 1).

**Table 2:** Test for positive selection and identification of positively selected sites

Species	Substitution rate		ω = d_*N*_/d_*S*_	Model [100]	2(L_b_-L_a_)	*P*	Positively selected sites								
			
	d_*N*_	d_*S*_					26	**37**	57	**60**	**70**	**71**	**74**	77	**86**
*G. microtarsus*	0.159	0.178	0.893	M1 – M2a	63.46	<0.001	**x**	**x**	**x**	**x**	**x**	**x**	**x**	**x**	**x**
	(± 0.028)	(± 0.039)	n.s.	M7 – M8	56.01	<0.001	**x**	**x**	**x**	**x**	**x**	**x**	**x**	**x**	**x**
				Dist. to human ABS			2	0	1	0	0	0	0	1	0
*M. incanus*	0.143	0.183	0.781	M1 – M2a	13.32	<0.005		**x**				**x**			
	(± 0.026)	(± 0.047)	n.s.	M7 – M8	11.50	<0.005					**x**	**x**			
				Dist. to human ABS				0			0	0			

The rates of non-synonymous (d_*N*_) and synonymous (d_*S*_) substitutions were calculated for the entire sequence. In the analyses of positively selected sites, twice the difference in likelihood 2(L_b_-L_a_) between the models was compared with a τ^2^-distribution (df = 2) to assess significance [100]. **x **represents sites under positive selection identified at the >95% confidence level. Sites in bold are antigen binding sites (ABS) according to the human DR1 molecule [37], distance (dist.) to human ABS corresponds to the amino acid distance between identified site and the nearest human ABS.

Positive selection on specific codon sites was detected using the maximum likelihood method CODEML implemented in PAML3.15 [39]. Two pairs of models were applied: M1a versus M2a, and M7 versus M8. The pair M1a – M2a has limitations in the presence of recombination, while M7 – M8 is robust against the effects of recombination [[40], but see [41]]. The models M2a and M8 that allow for positive selection fitted our data significantly better than the null hypothesis models M1a and M7 (Table 2). In *G. microtarsus*, both models M2a and M8 identified nine sites under positive selection, which were detected by Bayesian analysis (Table 2). Six of these are congruent with ABS from the human DR1 molecule [37], two are located directly next to an ABS, and another one at a distance of two amino acids from an ABS. For *M. incanus *only three positions were found to be under positive selection (Table 2). One position was detected by both models M2a and M8, and the two other positions were each detected by only one of the models. All three sites are congruent with human ABS.

### Test for gene conversion and recombination

Within the 44 MHC-DAB alleles of *G. microtarsus *the GENECONV analyses [42] detected no fragment significantly involved in gene conversion events in a global comparison (based on the whole alignment), but two fragments in the eight alleles of *M. incanus*. In pairwise tests, 98 fragments in *G. microtarsus *and two additional fragments in *M. incanus *were discovered but did not withstand corrections for multiple comparisons. However, in both species the numbers of pairwise internal fragments exceeded the random-assumption of 5% (*G. microtarsus*: 98 out of 946 (= 10.3%); *M. incanus*: four out of 28 comparisons (= 14.3%)) suggesting the occurrence of gene conversion in both taxa.

Population recombination was estimated using the software LDhat [43]. For *G. microtarsus*, the population recombination rate was high (ρ = 26) and deviated significantly from the null hypothesis (ρ = 0) in the likelihood permutation test. Here, the presence of recombination was confirmed while in *M. incanus *population recombination rate was quite low (ρ = 3) and, hence, the null hypothesis was not rejected.

The Hudson four-gamete test [44] implemented in DnaSP [45] revealed 21 and nine recombination events (R_M_) in *G. microtarsus *and *M. incanus*, respectively. These values indicate the minimum number of recombination events in the history of the samples.

### Evolutionary pattern of the MHC-DAB lineages

**Figure 2:**
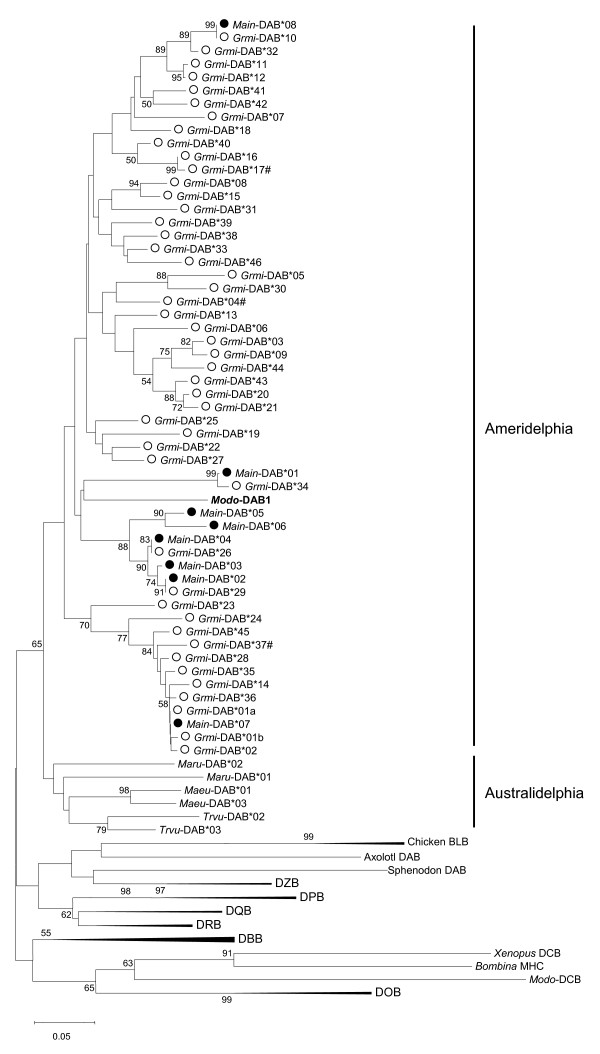
**Phylogenetic tree of the marsupial DAB locus in relation to the other MHC II β gene families**. The loci DOB, DPB, DQB, DRB are eutherian MHC class II β gene families, DZB is the prototherian family, and DBB and DCB are additional marsupial β gene families. Some branches have been compressed for a better overview. The tree was constructed with the neighbour joining method (Kimura-2-parameter), bootstrap values >50 are indicated (1,000 replications). The scale bar represents genetic distance in nucleotide substitution per site. # following an allele's name indicates a pseudogene. *Grmi *= *Gracilinanus microtarsus*, open circle, *Main *= *Marmosops incanus*, filled circle; *Modo = Monodelphis domestica*, in bold; *Maeu = Macropus eugenii*, *Maru = Macopus rufogriseus*, *Trvu = Trichosurus vulpecula*. Chicken = *Gallus gallus*, Axolotl = *Ambystoma mexicanum*, Xenopus = Xenopus laevis, *Bombina = Bombina bombina *and *Sphenodon = Sphenodon punctatus*.

To characterise the phylogenetic relationship of the marsupial DAB alleles found in this study a neighbour joining tree was constructed. It included the marsupial DAB, DBB and DCB loci, the prototherian DZB [46], representatives from the eutherian β gene families DOB, DPB, DQB and DRB as well as bird, reptile and anuran MHC sequences (Fig. 2). All MHC II β alleles discovered in this study clustered with published DAB sequences from other marsupial species (Fig. 2). The eight alleles of *M. incanus *did not form a monophyletic clade but were widely interspersed within the groups of *Grmi*-DAB alleles. Moreover, four *Main*-DAB alleles were found to be identical to alleles of *G. microtarsus*, at least for the 195 investigated base pairs. The identical allele pairs were: *Main*-DAB*02 and *Grmi*-DAB*29, *Main*-DAB*04 and *Grmi*-DAB*26, *Main*-DAB*07 and *Grmi*-DAB*01a, and *Main*-DAB*08 and *Grmi*-DAB*10. The Neotropical marsupials formed a sister group to the Australian marsupials, but without bootstrap support (Fig. 2). The entire DAB lineage was held together with a bootstrap support of 65, and separated from the other two known marsupial lineages DBB and DCB as well as from the eutherian and prototherian MHC II β gene lineages. However, the topology of β gene lineages was not supported by high bootstrap values.

## Discussion

Dramatic differences in the immune response between marsupials and eutherian mammals have been reported from earlier studies on marsupial immunology. For instance, *M. domestica *shows virtually no mixed lymphocyte response, which is a measure of T-cell function and highly dependent on MHC class II polymorphism [19-21]. The authors inferred that T-cell receptors are atypical and there is little or no polymorphism at the MHC class II genes in this species. A recent study by Siddle et al. [29] revealed a severely depleted MHC class I diversity in wild Tasmanian devils. The depleted MHC is probably accounting for the easy spread of the devil facial tumour disease, a contagious tumour that puts Tasmanian devils currently under the threat of extinction. In the present study, we investigated characteristic features of the MHC in free-ranging populations of Neotropical marsupials and tested whether the low levels of polymorphism at MHC class II in marsupials can be confirmed under natural selection conditions as inferred by earlier studies. Three different mechanisms for generating and maintaining MHC diversity were studied: positive selection, recombination and trans-species polymorphism.

In both study species (*Marmosops incanus*, *Gracilinanus microtarsus*) patterns of positive selection acting on the DAB loci were found by maximum likelihood analyses. Power and accuracy of the maximum likelihood method for detecting positive selection has been evaluated as strong by Wong and co-workers [47]. In these analyses ω was estimated not for the entire sequence, but separately for each codon site resulting in several significantly positively selected sites being detected [39]. There were nine positively selected sites in *G. microtarsus*; the human DR molecule contains 19 ABS in the corresponding segment [37]. Two ABS positions (65 and 68) out of those 19 human ABS were completely invariable in *G. microtarsus *suggesting that they are not functionally involved in antigen binding. In *M. incanus *only three positively selected sites were detected with two different models. We assume that only three positively selected sites were detected due to the low sample size of eight *Main*-DAB alleles. However, all of these three sites were congruent with human ABS and with positively selected sites in *G. microtarsus *emphasising structural similarities of sites probably involved in antigen binding throughout evolutionary lineages. This approach has recently been applied in MHC studies on wild populations of chamois [48,49], African mole-rats [50], voles [51], primates [52] and several salmonid species [53] to identify species-specific positively selected sites. All studies revealed high congruence of positively selected sites with the human ABS.

Intralocus recombination plays an important role in the generation of the large allelic polymorphism at the MHC [15,54,55]. Supporting results from free-ranging populations were provided by a number of studies [48,52,53,56,57]. In our study, we also found evidence for gene conversion and recombination in the history of the MHC-DAB alleles of the two marsupials although the tests we applied detected uneven numbers of events and probabilities. The program GENECONV was evaluated as having one of the highest probabilities of correctly inferring gene conversion events [58]. However, under extensive recombination the power of GENECONV might be reduced [51]. Richman et al. [15] showed that LDhat yields reliable estimates for the population recombination rate even when the amounts of both recombination and mutation are large, as is the case under balancing selection. The minimum number of recombination events calculated by DnaSP [45] showed a similar tendency as LDhat. The ambiguity in our results might be based on the presence of multiple loci in our data. We cannot discriminate between intra- and interlocus recombination as both mechanisms might interfere with each other in our dataset but it is assumed that interlocus recombination plays a minor role in MHC evolution of mammals [54].

Another indicator of balancing selection acting in a genetic system is trans-species polymorphism [18]. This non-neutral retention of alleles and lineages, across even multiple speciation events, is a typical feature of MHC evolution [3,17,18]. Patterns of this mode of evolution were described in a wide range of taxa including rodents [59-61], salmonids [53,62], ungulates [48,63], carnivores [64,65] and primates [66]. In our study we found MHC-DAB lineages shared between both species. But as we have shown that recombination is likely to have played a significant role in generating DAB diversity, conclusions about potential trans-species polymorphism should be treated cautiously. Recombination may have strong effects on phylogenetic inferences. For instance, it will cause allelic lineages appearing to have diverged over a longer time period than they actually did whereas the polymorphism as a whole is younger. This effect makes trans-species polymorphism more apparent [67]. Not only did we find shared lineages of MHC-DAB but also pairs of identical alleles, which constitute a more conclusive evidence of trans-species polymorphism. Trans-species sharing of MHC class II alleles between species of the same genus was documented in some studies on primates [68,69] but this phenomenon is rarely observed across genera. One striking example is the extensive MHC-DRB allele sharing between families of Malagasy lemurs [70].

In this study we are faced with multiple MHC-DAB loci, at least two in *M. incanus *and a minimum of four in *G. microtarsus*. A common problem in MHC-studies are difficulties in assigning alleles to their respective locus [51,71-73], which limits the significance of analyses on selection patterns. Different loci may be subjected to different selection pressures and processes and in a strict sense they cannot be combined in calculations on positively selected sites, trans-species polymorphism and sequence diversity. However, interlocus allelic exchange is known to occur at MHC loci [71], emphasizing its functional coherence. Also gene duplication is an important mechanism to generate polymorphism at the MHC, which is known, for instance, from rhesus macaques or Californian sea lions [74,75]. Despite this lack of knowledge, our study describing features of marsupial MHC diversity from natural populations is worthwhile even without locus assignment because it revealed results contradicting previous claims of low MHC class II variability in marsupials.

Our data on 54 free-ranging *G. microtarsus *disprove the previous assumption of very limited polymorphism at the MHC class II. *G. microtarsus *shows extensive polymorphism in the number of alleles within an individual and within the total sample as well as in the genetic distance among alleles. This level of variation is typical for classical MHC loci in studies on natural populations of numerous eutherian mammals. The evaluation of large genetic distance among the alleles should, however, be treated with caution, because we cannot discriminate between intra- and interlocus distances. At the current state of this work, tissue samples providing sufficient amounts of RNA are not available and thus we have no information on expression patterns of MHC class II in the investigated species.

In *G. microtarsus *one to seven alleles per individual were detected via cloning and sequencing. In preparation for this study, extensive sequencing revealed that 15 sequenced clones per individual provide a good estimate for allelic diversity [see Additional File 1]. Our results may still represent an underestimation of individual allelic diversity because the number of alleles detected per individual is correlated with the number of clones sequenced. However, we do not assume that low numbers of detected *Grmi*-DAB alleles in some individuals result from deficient sequencing, but from the presence of null alleles and/or a varying number of MHC-DAB loci in *G. microtarsus*. In individuals with one to three *Grmi*-DAB alleles some loci may have been missed by our primers or they simply do not exist. In some species the number of MHC class II loci varies between populations or even individuals of the same population. In cichlid fishes, for instance, the number of MHC class II B loci varies between haplotypes and individuals, ranging from one to thirteen loci [76]. In voles Bryja et al. [51] found that individuals differ in the number of DQA loci, showing either alleles from one or from two loci. Also for humans and other primates the number of DRBgenes present per haplotype can vary, it ranges from one to four in humans and from two to seven in the rhesus macaque [75,77].

*M. incanus *shows a similar dimension of MHC II diversity at the individual level, with each individual carrying 2–4 DAB alleles. In contrast, the number of eight MHC-DAB alleles in the total sample of 56 individuals is low (more than five times reduced compared to *G. microtarsus*) resulting in similar genotypes throughout the sample. Each individual revealed two to four *Main*-DAB alleles, which is in full concordance with the assumption of two loci in this species. Still the presence of null alleles cannot be excluded.

In general, low MHC variation in a population can be explained by relaxed selection pressure through pathogens and/or genetic drift in small populations, e.g. through a bottleneck [17,78] or by the mating system [79]. Diminished MHC diversity is demonstrated, for instance, in fin whales (*Balaenoptera physlaus*), sei whales (*B. borealis)*, Northern elephant seals (*Mirounga angustirostris*) [78,80], Malagasy giant jumping rats (*Hypogeomys antimena*) [79], moose (*Alces alces*) [81,82], common hamsters (*Cricetus cricetus*) [83] and in Scandinavian beavers (*Castor fiber*) [71].

The two marsupial species live sympatricly in the Atlantic rainforest of Brazil. They inhabit the same type of habitat, although *M. incanus *frequently uses the forest ground and the lower strata [84,85], while *G. microtarsus *is mainly arboreal and uses the canopy [86]. It was shown that both species differ in their microhabitat preferences and in their sensitivity to forest fragmentation [34,35]. Fragmentation does not negatively affect *G. microtarsus' *abundances, and it prefers more disturbed forest habitats. *M. incanus*, on the other hand, responds sensitively to forest fragmentation with decreasing abundances in small and isolated areas and increased abundance in old and less disturbed forests [34,35]. Males of both species are at least partially semelparous, meaning that they contribute to only one breeding season with multiple copulations and die afterwards due to severe physiological stress [87,88]. The females may survive and reproduce again, often in two or exceptionally in three consecutive seasons.

Hence, a reduced exposure to pathogens due to a monogamous mating system or a different environment can be excluded as an explanation of the low MHC diversity in *M. incanus *compared to *G. microtarsus*. Moreover, *M. incanus *should harbour more parasite infections compared to *G. microtarsus *because the mode of terrestrial locomotion increases the risk of parasitic infections through greater exposure to soil borne or faecal-orally transmitted parasites [89]. In fact Püttker et al. [90] reported from the same study populations a much higher nematode prevalence in *M. incanus *than in *G. microtarsus*. We assume that the explanation for the reduced MHC diversity in *M. incanus *might be a genetic bottleneck in the investigated population and that the diminished MHC diversity reflects a genome-wide loss of diversity. This explanation is supported by the fact that *M. incanus *is sensitive to habitat fragmentation, which probably led to reduced migration and isolation of the study population. *G. microtarsus *on the other hand is ecologically more flexible and non sensitive to fragmentation. Probably migration processes are less or not restricted and therefore MHC diversity is high. The pattern of low MHC diversity in *M. incanus *resembles several examples from eutherian mammals that revealed low MHC variation in populations after a genetic bottleneck [summarized in [5]].

In future analyses we aim to combine parasitological and genetic data on adaptive and overall neutral variability to investigate the role of forest fragmentation in shaping the different patterns of MHC diversity in the two marsupials. Lastly, expression analyses are required to shed light onto the functional differences in the MHC constitution of these two species.

## Conclusion

Despite the previous assumption that marsupials lack polymorphism at the MHC class II, our study revealed high levels of diversity in a free-ranging population of a South American marsupial. We tested for three different mechanisms that may generate and maintain MHC diversity: positive selection, recombination and trans-species polymorphism. The presence of all three mechanisms was confirmed. Moreover, typical large sequence divergence of the MHC alleles was found in both species. Beyond that, the two investigated marsupial populations revealed considerable differences in their MHC-DAB diversity. The processes leading to these differences will be investigated in future analyses including parasitological data and expression analyses.

## Methods

### Sampling

54 individuals of the Brazilian gracile mouse opossum (*G. microtarsus*) and 56 individuals of the Gray slender mouse opossum (*M. incanus*) were captured in forest fragments of the Brazilian Atlantic rainforest in the state of São Paulo, about 60 km south-west of the city of São Paulo. Animals were live-trapped and anesthetized (Forene, Abbott GmbH, Wiesbaden, Germany) for 1–2 minutes to allow small tissue samples to be taken from the ear, which were then stored in 70% ethanol. Subsequently, animals were released at their respective trapping location. For a detailed description of the study area, sites and trapping procedures see Püttker et al. [90,91].

### PCR and SSCP

DNA was extracted from ear tissue using the DNeasy Tissue Kit (Qiagen, Hilden, Germany). We examined a 195 bp fragment of the marsupial MHC class II DAB molecule coding for the major part of the β1 domain. This part contains most of the functionally important antigen binding sites in the β chain of the human DR1 molecule [37]. The fragment was amplified using primers JS1 (5'-GAG TGT CAT TTC TAC AAC GGG ACG-3') [92] and ML8 (5'-ACG CGC CTG CGC ACT AAG AAG GGC TC-3'). JS1 was previously designed for the mouse lemur *Microcebus murinus *and was successfully applied to several rodent species [71,93-95]. We designed ML8 for this study based on the DAB sequence of *M. domestica *(*Modo*-DAB1, accession No. AF010497).

PCR was conducted in a total reaction volume of 20 μl including 30–100 ng DNA, 0.375 μM of each primer (Sigma-Aldrich, Steinheim, Germany), 1× reaction buffer (10 mM TrisHCl, 50 mM KCl, 0.1% Triton ×100, 0.2 mg/ml BSA), 0.175 mM dNTPs and 1 unit of *Taq *polymerase (MPBiomedicals, Heidelberg, Germany). Thermocycling comprised an initial denaturation step at 95°C for 5 min, followed by 35 cycles of 30s denaturation at 95°C; 60s annealing at 56°C for *G. microtarsus *or 51°C for *M. incanus*, and 60s extension at 72°C. A final extension step was performed at 72°C for 10 min.

*M. incanus *individuals were genotyped via single strand conformation polymorphism (SSCP) [96]. For denaturation 1–4 μl PCR product were mixed with 6 μl loading dye (50 mM NaOH + 1 mM EDTA, Xylencyanol) and heated for 10 min at 50°C. The samples were subjected to electrophoresis on a 15% non-denaturing polyacrylamid gel (ETC, Kirchentellinsfurt, Germany). The best separation of MHC alleles was achieved by setting 200 V, 10 mA, 10 W for 20 min followed by 3 h at 450 V, 30 mA, 20 W (all steps conducted at constant 12°C). Gels then were fixed and silver stained (Plus One DNA Silver Staining Kit, GE Healthcare, Munich). The single strand patterns were excised from the gel matrix, eluted in 1 × TBE, re-amplified as described above for PCR and prepared for sequencing. Two alleles (*Main*-DAB*03 and *Main*-DAB*04) differed in two nucleotide but revealed identical SSCP patterns. Individuals with this pattern were labelled as *Main*-DAB*03/4 if their band patterns were not sequenced.

Due to a large number of MHC alleles per individual in *G. microtarsus *full separation of band patterns could not be accomplished through SSCP. *G. microtarsus *was examined therefore via molecular cloning after initial SSCP trials.

### Cloning and Sequencing

Molecular cloning often leads to an increase in misincorporation errors, therefore PCR products for cloning were generated with a proofreading polymerase (Hotstar Hifidelity polymerase, Qiagen, Hilden, Germany). Moreover, in heterogeneous templates (such as MHC genes) PCR amplifications are susceptible to the creation of artificial chimeric molecules from two related sequences [97]. The probability of chimeric molecule formation was reduced by two modifications of the PCR protocol described above: The number of cycles was reduced to 32 and the extension time prolonged to 2:30 min [97]. PCR products were purified (Cycle pure, Peqlab, Erlangen, Germany) and cloned into a pCR^®^4-TOPO vector using the TOPO TA cloning kit for sequencing (Invitrogen, Karlsruhe, Germany). At the beginning of the study we determined the required number of clones to be sequenced to reflect the individual MHC-diversity by extensive sequencing in two individuals [see Additional File 1]. Fifteen recombinant clones per sample were selected, PCR-amplified using the vector-primers T7 and M13rev, and purified. Sequencing and sequence analysis was carried out on an ABI PRISM 310 Automated Genetic Analyzer (Applied Biosystems, Foster City, Ca, USA) using the BigDye Terminator v3.1 Cycle Sequencing Kit (ABI).

A clone sequence was accepted as a MHC-DAB allele if one of the following criteria were met: (1) occurrence in two independent PCR reactions, (2) confirmation by SSCP or (3) a minimum of two positions differing from a known allele. Single sequences differing by one nucleotide from a known allele were attributed to the polymerase misincorporation error (declared by the company: 2.3 × 10^-6 ^per base per cycle, under our conditions one misincorporation every 70 molecules). Sequences differing in one position were observed more frequently than explained by this error, but we decided to follow a conservative approach so as to avoid overestimation of allelic diversity.

Sequences that were not confirmed by (1) or (2) were checked visually for PCR-mediated chimeric formations. Sequences composed of more than two fragments were assumed to be true recombination events because a multiple template switching during PCR is highly improbable. However, no PCR-mediated chimeric sequence was detected in our data. The MHC-DAB alleles of *G. microtarsus *and *M. incanus *were denominated as *Grmi*-DAB and *Main*-DAB corresponding to the MHC nomenclature suggested by Klein and co-workers [36].

### Test for positive selection

Under balancing selection a relative excess of non-synonymous over synonymous substitutions is expected [7,8]. We calculated the relative rates of synonymous (d_*S*_) and non-synonymous (d_*N*_) substitutions following the method of Nei and Gojobory [98] with the Jukes-Cantor [99] correction for multiple substitutions implemented in MEGA 3.1. The ratio ω = d_*N*_/d_*S *_was tested for significant deviation from one using a Z-test.

We used CODEML integrated into the software PAML 3.15 [39] to identify and test for positively selected codon sites (d_*N*_/d_*S *_> 1) in the MHC-DAB sequences. This was done separately for the two marsupial species. Based on maximum likelihood procedures the program estimates heterogeneous ω (= d_*N*_/d_*S*_) ratios among sites applying different models of codon evolution. The models are used to detect positively selected codon sites, and evaluated using a likelihood-ratio test (LRT). In the LRT two nested models are compared and the better fitting one is detected. Therefore, a model based on the null hypothesis of no selection is applied as well as a more complex model that assumes positive selection. The LRT is a comparison of twice the log-likelihood difference (2 [Lb – La]) with a τ^2^-distribution with the degrees of freedom equal to the difference in number of estimated parameters between the two models [100]. In this study, the models M1a (nearly neutral), M2a (positive selection), M7 (β) and M8 (β + ω) were employed [100]. Null model M1a assumes two site classes in the molecule with 0 < ω_0 _< 1 and ω_1 _= 1 in proportions *p*_0 _and *p*_1 _= 1 - *p*_0_. The alternative model M2a incorporates another class of sites with ω_2 _> 1 and the proportion *p*_2 _estimated from the data. Null model M7 assumes a beta distribution for ω, not allowing for positive selection (0 < ω < 1). In the alternative model M8 a third class of sites is added, which allows for positive selection (ω > 1) [101]. Anisimova et al. [40] showed that the combination of models M7 and M8 is robust against impact of recombination [41]. In the models M2a and M8 the probabilities for the site classes have been calculated by the Bayes empirical Bayes method (BEB) [101]. A site class with a mean ω > 1 is likely to be under positive selection.

### Test for gene conversion and recombination

The program GENECONV version 1.81 was used to detect sequence fragments that were likely to have been subjected to gene conversions. GENECONV detects pairs of sequences that share unusually long stretches of similarity given their overall polymorphism [102]. We used global and pairwise permutation tests (10,000 replicates) for both species separately to assess significance. No mismatches were accepted and p-values were corrected for multiple comparisons. In general, this approach is considered to be a powerful way of inferring recombination events correctly [58], but under extensive recombination as present at some MHC loci the power of GENECONV might be reduced [51].

We further applied the composite-likelihood method after Hudson [103] implemented in the program LDhat [43] to estimate the population recombination rate (ρ = 4N_e_*r*). The population recombination rate is a product of the crossing over rate per generation, *r*, and the effective population size, N_e_. This product can be estimated without prior information on these parameters [43]. LDhat works efficiently for sequences evolving under balancing selection even in the presence of numerous recombination events [104]. In a likelihood permutation test the population recombination estimate ρ was tested for statistical significance against the hypothesis of no recombination (ρ = 0). The minimum number of recombinant events (R_M_) was calculated after Hudson and Kaplan [four-gamete method, [43]] using the software DnaSP [45].

### Phylogenetic analyses

Sequence alignment and manual revision were performed in MEGA version 3.1 [105]. The same software was applied for the construction of a phylogenetic tree using the neighbour joining method [106], based on Kimura's two-parameter evolutionary distances [107]. In the phylogenetic tree the following Genbank sequences were applied: Marsupialia: *Macropus eugenii*: *Maeu*-DAB1 (AY438042), *Maeu*-DAB3 (AY856412), *Maeu*-DBB1 (AY438038), *Maeu*-DBB2 (AY438039), *Maeu*-DBB4 (AY438041); *Macropus rufogriseus: Maru*-DAB1 (M81624), *Maru*-DAB2 (M81626), *Maru*-DBB1 (M81625); *Monodelphis domestica: Modo*-DAB*01 (AF010497), *Modo*-DBB1 (XM_001369154), *Modo*-DBB2 (XM_001376782), *Modo*-DCB (XM_001376662); *Trichosurus vulpecula: Trvu*-DAB*02 (AF312029), *Trvu*-DAB*03 (AF312030), *Trvu*-DBB (AY271265). Monotremata: *Tachyglossus aculeatus: Taac*-DZB1 (AY288075); *Ornithorhynchus anatinus: Oran*-DZB1 (AY288074). Eutherians: *Aotus nancymaae*: *Aona*-DPB1 (AF486448); *Bos taurus*: *Bota*-DOB (AB117945); *Canis*: *Cafa*-DQB1 (NM_001014381); *Calu*-DOB (NM_001048127); *Felis catus*: *Feca*-DRB1 (AJ428212); *Homo sapiens: Hosa*-DRB (AM419948), *Hosa*-DPB1 (NM_002121), *Hosa*-DOB (NM_002120); *Macaca fascicularis*: *Mafa*-DPB1 (AB235893). *Sus scrofa*: Susc-DRB (AY135583), Susc-DQB (AY135571). Chicken: GagaBLB1 (NM_001044694), GagaBLB2 (M29763), GagaBLB3 (M26307); *Xenopus *XelaB3 (D13685); *Bombina bombina*: Bobom-Beta1-3 (EF210770), Bobom-5Race-1 (EF210766) and Bobom-3Race-1 (EF210761); *Ambystoma mexicanum*: *Amme*-DAB (AF209115); *Sphenodon punctatus*: *Sppu *DAB (DQ124232).

## Authors' contributions

TP and YML collected the field data. YML and CO carried out the molecular genetic analyses and managed the data. Statistical analyses, interpretation of the data and writing of the manuscript were performed by YML. SS conceived the study and the design, initiated its key collaborations and helped in drafting the manuscript. All authors read and approved the final manuscript.

## Supplementary Material

Additional file 1 Number of different MHC alleles in relation to the number of sequenced clones. Relationship between the number of sequenced clones and the number of different MHC-DAB alleles revealed in two individuals of *G. microtarsus *(a, b) and in two individuals of *M. incanus *(c, d). Click here for file
