# Supplementary material for: Selection, diversity and evolutionary patterns of the MHC class II DAB in free-ranging Neotropical marsupials
Source: BMC Genet. 2008 Jun 5;9:39. doi: 10.1186/1471-2156-9-39 (PMC2442840; doi:10.1186/1471-2156-9-39)
Supplement: Additional file 1 — Number of different MHC alleles in relation to the number of sequenced clones. Relationship between the number of sequenced clones and the number of different MHC-DAB alleles revealed in two individuals of G. microtarsus (a, b) and in two individuals of M. incanus (c, d). [file 1471-2156-9-39-S1.doc]

| a | b |
| --- | --- |
| c | d |
